# Diagnostic accuracy of rapid urine dipstick test to predict urinary tract infection among pregnant women in Felege Hiwot Referral Hospital, Bahir Dar, North West Ethiopia

**DOI:** 10.1186/1756-0500-7-481

**Published:** 2014-07-29

**Authors:** Tazebew Demilie, Getenet Beyene, Selabat Melaku, Wondewosen Tsegaye

**Affiliations:** 1 Department of Microbiology, Immunology and Parasitology, College of Medicine and Health Sciences, Bahir Dar University, P.O.Box 79, Bahir Dar, Ethiopia; 2 Department of Laboratory Sciences and Pathology, College of Public Health and Medical Sciences, Jimma University, P.O Box 378, Jimma, Ethiopia; 3 Microbiologist in Bahir Dar regional laboratory, Regional Health Research Laboratory, Microbiologist in Bahir Dar University, Bahir Dar, Northwest Ethiopia, Ethiopia

**Keywords:** Dipstick test, Pregnant women, UTI, Asymptomatic bacteriuria

## Abstract

**Background:**

Untreated bacteriuria during pregnancy has been shown to be associated with low birth-weight and premature delivery. Therefore, routine screening for bacteriuria is advocated. The decision about how to screen pregnant women for bacteriuria has always been a balance between the cost of screening versus the sensitivity and specificity. This study was designed to evaluate the diagnostic accuracy of the rapid dipstick test to predict urinary tract infection in pregnancy against the gold standard urine culture.

**Method:**

A total of 367 mid stream urine samples were collected, inoculated on MacConkey, Manitol salt agar (MSA) and blood agar and incubated aerobically at 37°C for overnight. Specimens were classified as “positive” for urinary tract infection (UTI) if the growth of the pathogen(s) was at a count ≥ 10^5^ colony-forming units per milliliter (cfu/mL) of urine and classified as “negative” with growth of <10^5^ cfu/mL. Urine samples were tested for the presence of nitrite and leukocyte esterase using dipstick rapid test in accordance to the manufacturer’s instructions.

**Results:**

From the total study participants, 37 pregnant women were symptomatic and the remaining 330 pregnant women were asymptomatic. The sensitivity and specificity of dipstick tests of leukocyte esterase was 50% and 89.1% for pregnant women with asymptomatic UTI(ABU) and 71.4% and 86.7% for symptomatic UTI respectively and for nitrite 35.7% and 98.0% for ABU and 57.1% and 96.7% symptomatic UTI.

**Conclusion:**

This study revealed that the use of dipstick leukocyte esterase and nitrite for screening UTI particularly asymptomatic bacteriuria was associated with many false positive and negative results when it was compared against the gold standard culture method. The low sensitivity and positive predictive value of urine dipstick test proved that culture should be used for the diagnosis of UTI*.*

## Background

Urinary tract infection (UTI) is an infection caused by the presence and growth of microorganisms anywhere in the urinary tract. It is usually happen due to bacteria from the digestive tract ascending the opening of the urethra and begin to multiply to cause infection [1,2]. Pregnant women are at increased risk for UTIs, beginning in week 6 and peaking during weeks 22 to 24, approximately 90 percent of pregnant women develop urethral dilatation, which will remain until delivery. Increased bladder volume and decreased bladder tone, along with decreased urethral tone, contribute to increased urinary stasis and ureterovesical reflux [3].

UTI can be either symptomatic or asymptomatic, and patients with significant bacteriuria who have symptoms referable to the urinary tract are said to have symptomatic bacteriuria. Asymptomatic bacteriuria (ABU) is a condition characterized by bacteriuria without classical symptoms attributable to the urinary tract [4]. The relatively high prevalence of UTI particularly asymptomatic bacteriuria during pregnancy, which will have significant consequences can be avoided with treatment if they are screened early for bacteriuria. The decision about how to screen asymptomatic women for bacteriuria has always been a balance between the cost of screening versus the sensitivity and specificity [5]. In addition, the correctness of the laboratory test in determining the individual status is crucial not to give false positive or negative results that may lead to unnecessary prescription of drug or inappropriate management.

Even though there are various methods for screening, urine culture still remains the gold standard for diagnosis of UTI and has been advocated as a screening procedure for urinary tract infection in pregnancy. It is however, expensive, and takes 24–48 h to obtain result and requires skilled personnel. Unfortunately, appropriate investigations necessary for the diagnosis of urinary tract infection are not available in many health facilities [6].

Dipstick test is one of the qualitative diagnostic method used to detect UTI and have the advantage of being easy to perform, interpret, can be carried out in primary care giving facilities and result can be obtained immediately [7]. Though evaluation of dipstick test has been conducted in different countries(6,7), it has never been tested in the northern part of Ethiopia around Bahir Dar and information generated from this study will help clinician and laboratory technologist for the decision to determine to use culture and dipstick test in accordance their relevance. Thus, the objective of this study was to evaluate the diagnostic accuracy of the rapid dipstick test to predict urinary tract infection in pregnancy in comparison with the gold standard of urine culture.

## Methods

A hospital based prospective cross sectional study was conducted at Felege Hiwot Referral Hospital (FHRH) from October 2010 to January 2011 to evaluate the diagnostic accuracy of the rapid urine dipstick test to predict urinary tract infection in pregnancy with age group ranged from 17 to 40 years with gestation period of 16 to 38 weeks coming for prenatal follow up.

A pre-designed and structured questionnaire was used for the collection of data on socio-demographic characteristics. The presences of symptoms of UTI were checked by Gynaecologists and senior nurses.

A total of 367 pregnant women (37 with complaints and 330 without complaints of UTI) were participated in the study and pregnant women who have taken antibiotics within seven days at the time of recruitment and who were not willing to participate were excluded from this study.

Symptomatic UTI refers to patients whose urine is yielding positive culture (≥10^5^ cfu/ml) and who have symptoms referable to the urinary tract infection, where as asymptomatic bacteriuria (ABU) refers to pregnant women whose two consecutive urine samples showed positive cultures (≥10^5^ cfu/ml) of the same uropathogen and without showing sign and symptoms of UIT [8].

### Specimen collection and processing

To reduce the risk of contamination, pregnant women were informed to clean their hands with water and their genital area with swab soaked in normal saline before collection of the clean catch mid stream urine samples.The samples were collected using sterile, wide mouthed glass bottles with screw cap tops and were processed in the laboratory within 2 hours of collection and specimens that were not processed within 2 hours were kept refrigerated at 4°C until it was processed.

### Dipstick test

From all participants urine samples were tested for the presence of nitrite and leukocyte esterase using dipstick rapid test. The Ames Multiple Reagent Strip (Bayer Diagnostics Australia) was used for all specimens in accordance with the manufacturer’s instructions. A positive dipstick was defined as the presence of nitrites or a reaction of greater than or equal to a trace of leukocytes. A negative dipstick was defined as the absence of any reaction for leukocytes and nitrites.

Urine dipstick bottles were stored at room temperature and expire dates also checked before use. Urine dipstick test results were interpreted according to the manufacturer’s instruction.

### Quantitative urine culture

Mixed freshly collected clean catch mid stream urine specimens were taken using a sterile calibrated wire loop (one that hold 1/500 ml or 0.002 ml) and were inoculated on a plate MacConkey (Oxoid, Ltd, England), Manitol salt agar (MSA) (Oxoid, Ltd, England) and blood agar (Oxoid, Ltd, England) and were incubated aerobically at 37°C for overnight. Specimens were classified as “positive” for UTI if the yielded of growth of a pathogen(s) was at a count ≥ 10^5^ colony-forming units per milliliter (cfu/mL) of urine. Specimens were classified as “negative” if culture showed growth of <10^5^ cfu/mL and considered as contaminated, when mixed bacterial growth was observed [2,8].

Standard reference strains, *Staphylococcus aureus* (ATCC25923), *Escherichia coli* (ATCC25922 and *P. aeruginosa* (ATCC 27853) were used to assure testing of quality of culture media.

### Data entry and analysis

All data were entered into the computer and analyzed using the EPI Info Software. The degree of correlation between the components of the dipstick singly and in various combinations was compared against urine culture result. The comparative diagnostic value of the various components of the dipstick test and culture were evaluated in terms of sensitivity, specificity, positive predictive value (PPV) and negative predictive value (NPV) against urine culture (gold standard).

### Ethical consideration

The study was conducted after getting a full approval from Jimma University post graduate and research coordinating office, Amhara national regional state health bureau and FHRH. In addition written informed consent for the study was obtained from the study participants and confidentiality of results was kept. All dipstick and culture results were sent to the responsible physicians as soon as possible so that the pregnant women were directly benefited from the study.

## Results

A total of 367 pregnant women with and without symptoms of urinary tract infection (UTI) were investigated during the study period. The minimum and the maximum age of the pregnant women were 17 and 39 years respectively with mean age was 25.8. The predominated age group was 20–24 years (43.9%), the majority group of study participants are at their second (39.1%) and third (47.0%) stage of pregnancy with no significance variation with the rate of bacteriuria . Educational status of participants varied from illiterate to postgraduate studies, 66 (18%) of the respondents were unable to read and write while 25.1% of the participants had higher education (Table 1).

**Table 1:** Socio-demographic characteristics of study participants in Felege Hiwot Referral Hospital (FHRH), Bahir Dar, October 2010-January 2011

**Variables**	**Number**	**Percent**
**Age**		
15-24	200	54.5
25-34	159	43.3
35-44	8	2.2
**Gestation stage**		
First trimester	49	13.4
Second trimester	141	38.4
Third trimester	177	48.2
**Marital status**		
Married	334	99.2
Others	3	0.8
**Occupation**		
Employed	110	30
Unemployed	257	70
**Residence**		
Urban	354	96.5
Rural	13	3.5
**Religion**		
Orthodox	321	87.5
Muslim	33	9
Protestant	13	3.5
**Ethnicity**		
Amhara	357	97.3
Oromo	5	1.4
Tigray	3	0.8
Other	2	0.5
**Education**		
Illiterate	66	18
Read and write	12	3.3
Elementary	67	18.3
High school	130	35.4
Higher education	92	25.1
**Income/month**		
<500	53	14.4
501-1000	96	27.5
1001-1500	63	17.4
1501-2000	55	15.0
>2000	100	28.3
**Total**	**367**	

Out of the total 367 study participants, 330 pregnant women were without sign and symptoms for UTI and were screened for asymptomatic bacteriuria (ABU). The dipstick rapid test result showed that 55 (15%) of the pregnant women were positive for leukocyte esterase and 21 (5.7%) pregnant women were positive for nitrite (Table 2).

**Table 2:** Dipstick test result for leukocyte esterase and nitrite in relation with culture result of ABU, symptomatic UTI and overall UTI in FHRH, Bahirdar, October 2010-January 2011

**Type of UTI**	**Dipstick tests**		**Culture**			**Sensitivity**	**Specificity**	**PPV**	**NPV**
			**Positive N**^**o **^**(%)**	**Negative N**^**o **^**(%)**	**Total N**^**o **^**(%)**				
**ABU**	**LE**	Positive	14(29.9)	33(70.2)	47(14.2)	50.0%	89.1%	29.8%	95.1%
		Negative	14(5)	269(95)	283(85.8)				
		Total	28(8.5)	302(91.5)	330(100)				
	**Nitrites**	Positive	10(62.5)	6(37.5)	16(4.9)				
		Negative	18(5.7)	296(94.3)	314(95.1)	35.7%	98%	62.5%	94.3%
		Total	28(8.5)	302(91.5)	330(100)				
**Symptomatic UTI**	**LE**	Positive	5(62.5)	3(37.5)	8(21.6)				
		Negative	2(6.9)	27(93.1)	29(78.4)	71.4%	90.0%	62.5%	93.1%
		Total	7(18.9)	30 (81.1)	37(100)				
	**Nitrites**	Positive	4(80)	1(20)	5(13.5)				
		Negative	3(9.4)	29(90.6)	32(86.5)	57.1%	96.7%	80.0%	90.6%
		Total	7(18.9)	30(81.1)	37(100)				
**Overall UTI**	**LE**	Positive	18(32.7)	37(67.3)	55(15.0)				
		Negative	17(5.5)	295(94.5)	312(85)	51.4%	88.9%	32.7%	94.5%
		Total	35(9.5)	332(90.5)	367(100)				
	**Nitrites**	Positive	15(71.4)	6(28.6)	21(5.7)				
		Negative	20(5.8)	326(94.2)	346(94.3)	42.9%	98.2%	71.4%	94.2%
		Total	35(9.5)	332(90.5)	367(100)				
	**LE or Nitrites**	Positive	25(71.4)	10(26.6)	35(9.5)				
		Negative	10(3.0)	322(97)	332(90.5)	71.4%	97.0%	71.4%	97.0%
		Total	35(9.5)	332(90.5)	367(100)				
	**LE and nitrites**	Positive	8(72.7)	3(27.3)	11(3.0)				
		Negative	27(7.6)	329(92.4)	356(97)	22.9%	99.1%	72.7%	92.4%
		Total	35(9.5)	332(90.5)	367(100)				

key: ABU: Asymptomatic Bacteruria, LE: Leukocyte Esterase, NPV: Negative predictive value, PPV: Positive predictive value, UTI: Urinary tract infection.

From the 35 bacterial isolates (28 from ABU), 23 were Gram negative while 12 were Gram positive bacteria. Among the identified bacteria *E.coli* was the most common bacteria accounting for 16 (45.7%) isolates followed by Coagulase negative Staphylococcus (CNS) and *S.aureus* with isolation rate of 17.1% and 8.6% respectively, and only 2 (5.7%) *Proteus mirabilis* were indentified (data not shown).

Taking culture result as the gold standard, the sensitivity and specificity was 50% and 89.1%, and 35.7% and 98.0% for leukocyte esterase and nitrite respectively for asymptomatic UTI (Table 2).

When we consider both leukocyte esterase and nitrite dipstick test result together taking account positive results in either of the test, the sensitivity of dipstick was increased to 71.4% than using either of the tests alone. Where as if we take only positive cases for both tests the sensitivity were decreased to 22.9% than either of the tests alone.

## Discussion and conclusions

Routine screening pregnant women for bacteriuria advocated since untreated bacteriuria during pregnancy has been shown to be associated with low birth-weight and premature delivery [9].

In our study the sensitivity and specificity of urine dipstick (nitrate and leukocyte esterase in combination) was 22.9% and 99.1% respectively which is comparable with the study done by Masinde *et al.*[10] in Tanzania, who reported the sensitivity and specificity of the dipstick test result 38.9% and 86.7% respectively. In the current study the sensitivity of leukocyte esterase and nitrite was 51.4% and 42.9%, while its specificity was 88.9% and 98% which has similar pattern with the study reported by Kacmaz *et al.*[6] where the sensitivity of urine leukocyte esterase and nitrite was found to be 70% and 60%, and specificity was 92.5% and 99.2% respectively.

In our study the sensitivity of leucocytes esterase for detection ABU was 50% where as the sensitivity for nitrate was 35.7% which is comparable with a study on diagnostic accuracy of dipstick for the diagnosis of ABU in Nigeria by Eigbefoh *et al.*[11] who reported that the sensitivity of leucocytes esterase for detecting ABU was 56.8% and the sensitivity of nitrite was 21.6%.

This study witnessed that sensitivity of urine dipstick for nitrite and leukocyte esterase slightly increases in symptomatic UTI than ABU. A study by Assefa *et al.*[12] also proved that percentage of significant pyuria slightly increase in symptomatic UTI than ABU. The sensitivity of leukocyte esterase and nitrite increase when taking positive result when one of the test was positive, while the sensitivity decrease when considering positive result when in both tests were positive. A study by Eigbefoh *et al.*[11] in Nigeria also reported decrease in the sensitivity of urine dipstick when both leukocyte esterase and nitrite test result were used together.

In Ethiopia the majority of health care facilities use dipstick analysis for nitrites and leukocyte esterase determination and microscopic examination for pyuria and bacteriuria for screening UTI (both ABU and symptomatic UTI) but according to different studies these dipstick tests have poor sensitivity and positive predictive values to detect bacteriuria especially in asymptomatic pregnant women [5,13,14]. In this study, it is also shown that urine dipstick testing for nitrites and leukocyte esterase can poorly detect all the culture positive bacteriuria cases in pregnant women. The low sensitivity of leukocyte esterase may be attributed to that the presence of leukocyturia does not always correlate with bacteriuria. Pyuria is not specific for UTI and may occur with other inflammatory disorders of the genitourinary tract (e.g., vaginitis) and infections with other uropathogens such as Chlamydia and *N.gonorrhoeae*. Moreover, the leukocyturia may continue even if the bacteriuria has cleared spontaneously or after treatment [6,15,16]. In contrast the low sensitivity of nitrite may be due to that nitrite test depends on the detection of nitrite in the urine which is formed from nitrate by many uropathogens. Positive nitrite test indicates that nitrite has been produced from the reduction of nitrate by enteric bacteria, most commonly by the genera of the *Enterobacteriaceae* family. The presence of nitrite is highly specific for bacteriuria, but several uropathogens other than *Enterobacteriaceae* do not reduce nitrate to nitrite [6,17]. False negative results may occur when a UTI is caused by organism that do not contain nitrite reductase, when the urine has been in the bladder for insufficiently long periods for the reduction of nitrite to occur or when dietary nitrite is absent. A negative test may also signify absence of nitrites in the urine due to a reduction of nitrates beyond the nitrite stage [6,14].

The use of dipstick leukocyte esterase and nitrite for screening UTI particularly with asymptomatic bacteriuria was associated with many false positive and negative results as compared to the gold standard culture method. Despite these limitations, a positive test suggestive of UTI for further evaluation of clinical manifestations for empirical treatment, while a negative test is an indication for urine culture. The urine dipstick test, if positive, will also be useful in follow-up of patient after treatment of urinary tract infection. This is useful in poor resource setting especially in the third world where there is a dearth of trained personnel and equipment for urine culture. Though this study proved the low sensitivity and specificity of the dipstick tests, the tests can be used to manage at least positive cases empirically.

## Competing interests

The authors declare that they have no competing interests.

## Authors’ contributions

DT was responsible for inception of the idea and write up of the proposal and conduct the laboratory work. BG was responsible in designing the study and drafting the manuscript, whereas TW was involved in the design of study the analysis and interpretation of the data, and correction of the final draft of the manuscript.MS was responsible for collection of samples and data. All authors read and approved the final manuscript.
